# Physiologically generated presenilin 1 lacking exon 8 fails to rescue brain *PS1−/−* phenotype and forms complexes with wildtype PS1 and nicastrin

**DOI:** 10.1038/srep17042

**Published:** 2015-11-26

**Authors:** Hannah Brautigam, Cesar L. Moreno, John W. Steele, Alexey Bogush, Dara L. Dickstein, John B.J. Kwok, Peter R. Schofield, Gopal Thinakaran, Paul M. Mathews, Patrick R. Hof, Sam Gandy, Michelle E. Ehrlich

**Affiliations:** 1 Fishberg Department of Neuroscience and Friedman Brain Institute, Icahn School of Medicine at Mount Sinai, New York, NY 10029; 2 Department of Neurology, Icahn School of Medicine at Mount Sinai, New York, NY 10029; 3 Department of Pediatrics, Icahn School of Medicine at Mount Sinai, New York, NY 10029; 4 Department of Psychiatry, Icahn School of Medicine at Mount Sinai, New York, NY 10029; 5 Alzheimer’s Disease Research Center, Icahn School of Medicine at Mount Sinai, New York, NY 10029; 6 Graduate School of Biomedical Sciences, Icahn School of Medicine at Mount Sinai, New York, NY 10029; 7 Ronald M. Loeb Center for Alzheimer’s Disease, Icahn School of Medicine at Mount Sinai, New York, NY 10029; 8 School of Pharmacy, University of Wisconsin-Madison, Madison, WI 53706; 9 James J. Peters VA Medical Center, Bronx, NY 10468; 10 Department of Cellular and Molecular Medicine,University of California San Diego, La Jolla, CA 92037; 11 Department of Preclinical Biology, OrPhi Therapeutics, Carlsbad, CA 92008; 12 Department of Physiology, Perelman School of Medicine, University of Pennsylvania, PA 19104; 13 Neuroscience Research Australia, Sydney, NSW 2031, Australia; 14 School of Medical Sciences, University of New South Wales, Sydney, NSW 2052, Australia; 15 Departments of Neurobiology, Neurology, and Pathology, The University of Chicago, IL 60637; 16 Department of Psychiatry, New York University School of Medicine, New York, NY 10016; 17 Nathan Kline Institute Center for Dementia Research, Orangeburg, NY 10962.

## Abstract

The presenilin 1 (*PSEN1)* L271V mutation causes early-onset familial Alzheimer’s disease by disrupting the alternative splicing of the *PSEN1* gene, producing some transcripts harboring the L271V point mutation and other transcripts lacking exon 8 (PS1^∆exon8^). We previously reported that PS1 L271V increased amyloid beta (Aβ) 42/40 ratios, while PS1^∆exon8^ reduced Aβ42/40 ratios, indicating that the former and not the exon 8 deletion transcript is amyloidogenic. Also, PS1^∆exon8^ did not rescue Aβ generation in PS1/2 double knockout cells indicating its identity as a severe loss-of-function splice form. PS1^∆exon8^ is generated physiologically raising the possibility that we had identified the first physiological inactive PS1 isoform. We studied PS1^∆exon8^ *in vivo* by crossing PS1^∆exon8^ transgenics with either PS1-null or Dutch APP^E693Q^ mice. As a control, we crossed APP^E693Q^ with mice expressing a deletion in an adjacent exon (PS1^∆exon9^). PS1^∆exon8^ did not rescue embryonic lethality or Notch-deficient phenotypes of PS1-null mice displaying severe loss of function *in vivo*. We also demonstrate that this splice form can interact with wildtype PS1 using cultured cells and co-immunoprecipitation (co-IP)/bimolecular fluorescence complementation. Further co-IP demonstrates that PS1^∆exon8^ interacts with nicastrin, participating in the γ–secretase complex formation. These data support that catalytically inactive PS1^∆exon8^ is generated physiologically and participates in protein-protein interactions.

Over 180 mutations in the presenilin-1 gene (*PSEN1*, PS1) have been identified, all leading to early-onset familial Alzheimer’s disease (FAD)^1^,^2^,^3^. *PSEN1* encodes a 9- or 10-transmembrane-domain PS1 protein that associates with nicastrin, APH-1, and PEN-2 to form the ~10^6^ Da γ-secretase aspartyl proteinase complex^4^. The primary function of PS1 is to form the catalytic site of the γ-secretase aspartyl proteinase complex, which is activated following endoproteolysis within its cytoplasmic loop and subsequent association of each N-terminal fragment (NTF) with its cognate C-terminal fragment (CTF)^5^,^6^,^7^,^8^.

Over the past decade, we have investigated the molecular mechanisms associated with a mutant PS1 lacking exon 8 (PS1^∆exon8^) that is generated by the L271V splice site mutation^9^. We identified this point mutation in a Tasmanian family (Tas-1) in 2003^9^. The cognitive deficits and disease progression fit the pattern of typical FAD^10^; however, late in the illness, all family members harboring the Tas-1 mutation exhibit myoclonus, a relatively uncommon motor manifestation of late AD^9^. Affected individuals exhibited large atypical “cotton wool”, non-cored plaques in the neocortex and hippocampus^9^.

We observed that some PS1^∆exon8^ molecules are apparently generated physiologically by normal wildtype (wt) cells at an estimated abundance of 5–15% of all PS1 transcripts in control neuroblastoma cells as well as in brain cortex from other FAD cases (without PS1 mutations)^9^. Others had reported that physiological expression of PS1^∆exon8^ splice variants was primarily restricted to leukocytes^11^. In Tas-1 family members harboring the L271V mutation, a 17–50% increase in transcripts lacking exon 8 was observed^9^. In cell culture studies, we noted that the pathogenic elevation in Aβ42/40 ratio was greater in association with the PS1 L271V point mutation than with the PS1^∆exon8^ deletion mutation. Thus, PS1 L271V − not PS1^∆exon8^ − is probably the FAD pathogenic, amyloidogenic species^9^.

Despite its exclusion as the pathogenic species underlying Tas-1 FAD, some novel properties made this physiologically generated alternative splice form an interesting focus for further study. Biochemical analysis revealed that PS1^∆exon8^ was unable to rescue Aβ generation by *PSEN*1/2 double knockout (DKO) cells, but in the presence of wt *PSEN*1/2, PS1^∆exon8^ caused an unusual perturbation of Aβ speciation^9^.

Based on its inability to rescue Aβ generation in *PSEN*1/2 DKO cells, PS1^∆exon8^ thus appeared to be a previously undocumented, physiologically generated, catalytically inactive splice form. Severe loss-of-function PS1 mutations, such as L435F, have recently been identified and characterized in cultured cells, but these have all been associated with pathogenicity for FAD^2^. In Tas-1 FAD, however, PS1^∆exon8^ is a physiologically generated alternative splice form that accumulates in excess. Yet, the contribution of PS1^∆exon8^ to disease pathogenesis, if any, is unknown. Understanding the mechanism of action of these unusual PS1 molecules is highly relevant because their effects in the presence of wt PS1/2 suggests an intermolecular communication *in trans* between PS1^∆exon8^ and wt PS1 that is not explicitly predicted by the current 1:1:1:1 stoichiometry model for the γ-secretase complex^12^. This *in trans* model has been recently proposed by Kelleher and colleagues in his characterization of a severe loss-of-function FAD mutant PS1^13^.

Because of this apparently unconventional behavior of PS1^∆exon8^, we formulated two hypotheses that we then set out to test. The first hypothesis was that PS1^∆exon8^ was truly inactive in brain *in vivo*. While one or two naturally occurring, severely hypomorphic PS1 mutations have been reported^2^, none have been tested *in vivo* to confirm the effect on the Notch-deficient phenotype that defines the *PS1−/−* mouse^14^. As the failure to rescue Notch processing is responsible for the most severe and defining features of the *PS1−/−* phenotype, we investigated whether PS1^∆exon8^ could rescue the lethal and/or brain phenotype of the PS1 KO mouse^15^. This report constitutes evidence that PS1^∆exon8^ is the first physiologically generated severely hypomorphic alternate splice form to be studied in this rescue paradigm.

The second hypothesis that we sought to test was that PS1^∆exon8^ participates in a physical interaction with wt PS1. At the time our study was reported^9^, the favored model for γ-secretase complex structure involved dimerization of two PS1 molecules, as described by Kopan and colleagues^4^. That model provided an obvious situation conducive to physical contact between wt and mutant PS1 molecules. However, with the more recent challenges to the Kopan PS1 dimer model of γ-secretase complex structure^4^ and the currently accepted 1:1:1:1 stoichiometry model^12^, an opportunity for *in trans* interaction between PS1^∆exon8^ and wt PS1 was obvious. It is worth noting that while the experiments reported herein were in progress, Kelleher *et al.* reported a severe loss-of-function point pathogenic mutation in PS1 that underwent *in trans* mutant PS1-wt PS1 interactions^13^. We employed a co-immunoprecipitation/bimolecular fluorescence complementation approach to demonstrate the existence of a physical interaction *in trans* involving PS1^∆exon8^ and wt PS1. With regard to the physical status of PS1^∆exon8^, we have established herein the ability of PS1^∆exon8^ to participate in protein-protein interaction with nicastrin, the first step involved in the formation of γ–secretase complexes. However, we cannot determine whether either, both, or neither of these interactors is incorporated into complexes when the interactions occurred.

## Results

### PS1^∆exon8^ does not rescue embryonic mouse PS1 KO lethality

**Figure 1:**
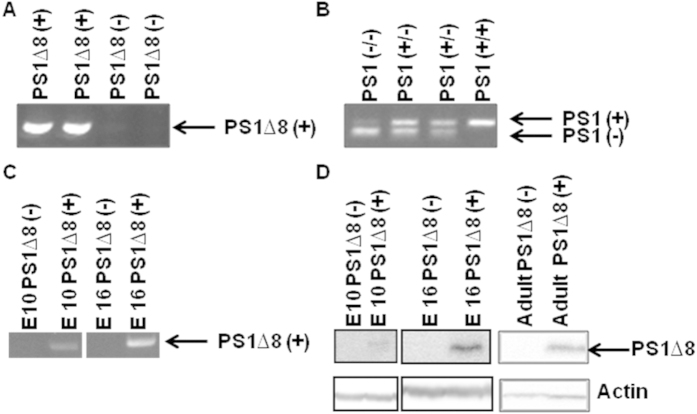
PS1^∆exon8^ is expressed as early as embryonic day 10. (**A**) Genotyping analysis of embryos by PCR for the human PS1^∆exon8^ transgene. (**B**) Genotyping results for murine PS1. The upper band corresponds to the PS1(+) allele while the bottom band corresponds to the mutant, KO allele. (**C**) PS1^∆exon8^ mRNA expression from embryonic mouse brain extracts by RT-PCR at E10 and E16. Expression is seen as early as E10. (**D**) Immunoblot of embryonic mouse brain extracts at E10 and E16 and adult mouse brain hippocampal extracts at 18 months of age using monoclonal human specific anti-N-terminus PS1, NT.1 antibody to confirm transgene protein expression. The bottom immunoblot is an actin loading control.

**Figure 2:**
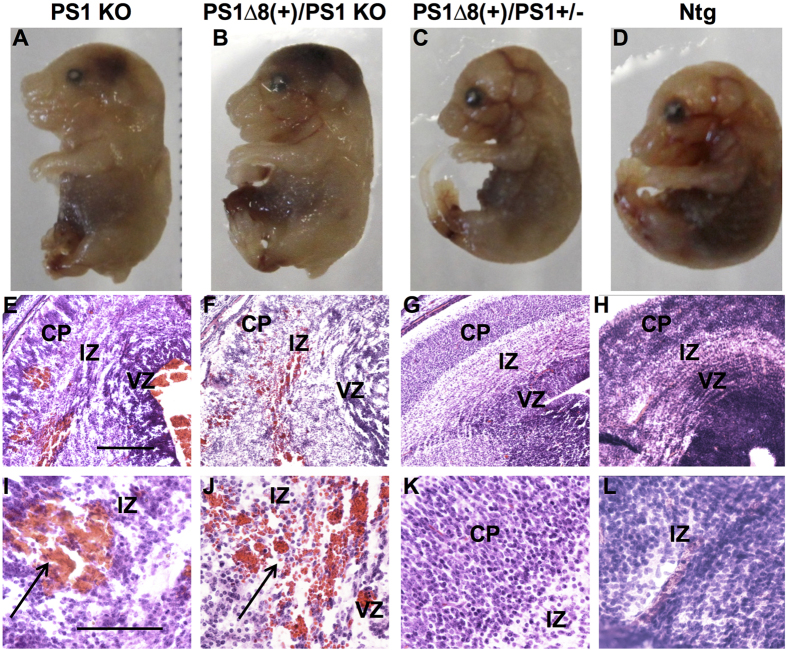
PS1^∆exon8^ does not rescue embryonic PS1 KO lethality. (**A**) E16 hPS1^∆exon8^(−)/mPS1 KO embryo exhibits a shortened rostro-caudal body axis and tail and intracranial hemorrhaging. (**B**) hPS1^∆exon8^(+)/mPS1 KO embryo resembles the hPS1^∆exon8^(−)/mPS1 KO embryo exhibiting a shortened rostro-caudal body axis and tail and intracranial hemorrhage. (**C**) One copy of murine PS1, hPS1^∆exon8^(+)/mPS1(+/−), results in normal development. (**D**) Ntg embryo shows normal development as well. Sections stained with hematoxylin-eosin from the embryos above show intracranial hemorrhaging in mPS1 KO ((**E**,**I**), higher magnification) and hPS1^∆exon8^(+)/mPS1 KO ((**F**,**J**), higher magnification), in the intermediate zone (IZ) next to the left lateral ventricle (arrow), while there is no detectable hemorrhage in hPS1∆8(+)/mPS1(+/−) ((**G**,**K**), higher magnification) or Ntg ((**H**,**L**), higher magnification). Scale bar in (**E**–**H**), 500  μm and (**I**–**L**), 200  μm. CP = cortical plate, IZ = intermediate zone, VZ = ventricular zone.

To confirm that deletion of exon 8 causes a loss of PS1 activity *in vivo*, we assessed whether PS1^∆exon8^ was able to rescue mouse PS1-null lethality. We hypothesized that if PS1^∆exon8^ were truly a complete loss-of-function mutation, PS1^∆exon8^ would not rescue PS1 KO lethality in embryonic mice. For the initial experiments, two hPS1^∆exon8^ (+/−) heterozygote mice were bred and 20 positive PS1^∆exon8^ transgenic mice were then individually bred to a C57Bl6/J mouse. None of the resulting 20 mice were homozygous, as each of those breedings produced Ntg mice. Also, viable mice with the hPS1^∆exon8^ (+/−)/mPS1(*−/−*) genotype were not produced from over 50 pups that were genotyped, and therefore E16-18 embryos from timed matings were examined. Human PS1^∆exon8^ expression from some of these initial breedings is shown in Fig. 1A. Also, each embryo was genotyped for the presence of the PS1^∆exon8^ transgene and endogenous PS1 (Fig. 1B). Brains from hPS1^∆exon8^ (+) pups were assayed for transgenic mRNA and exhibited expression as early as E10 (Fig. 1C). This unique band visible in hPS1^∆exon8^ mice, showed a clear increase in PS1^∆exon8^ expression from embryonic day 10 to 16, and expression remained stable throughout adulthood (Fig. 1C,D). At E16, mPS1 (*−/−*) (KO) embryos (Fig. 2A) were grossly indistinguishable from hPS1^∆exon8^(+/−)/mPS1(*−/−*) (KO) embryos (Fig. 2B). The mutant phenotype included a shortened rostro-caudal body axis, brain hemorrhage, and skeletal abnormalities. However, hPS1^∆exon8^(+/−)/mPS1(+/−) embryos carrying a single copy of mPS1, showed no abnormalities (Fig. 2C) compared to a mPS1(+/+) (Ntg) embryo (Fig. 2D). We confirmed the presence of hemorrhage in the intermediate zone near the lateral ventricle in the mPS1(*−/−*) KO embryo (Fig. 2E,I) and hPS1^∆exon8^ (+)/mPS1(*−/−*) embryo (Fig. 2F,J), while hPS1∆^exon8^(+) with a single endogenous mouse PS1 allele mPS1(+/−) (Fig. 2G,K) and the mPS1(+/+) (Ntg) embryos (Fig. 2H,L) showed no hemorrhage. To phenotype the adult transgenic mouse, we utilized mice carrying a single copy of hPS1^∆exon8^ on a mPS1(+/+) genetic background.

### PS1^∆exon8^ transgenes do not exacerbate motor deficits caused by FAD Dutch APP transgenes

**Figure 3:**
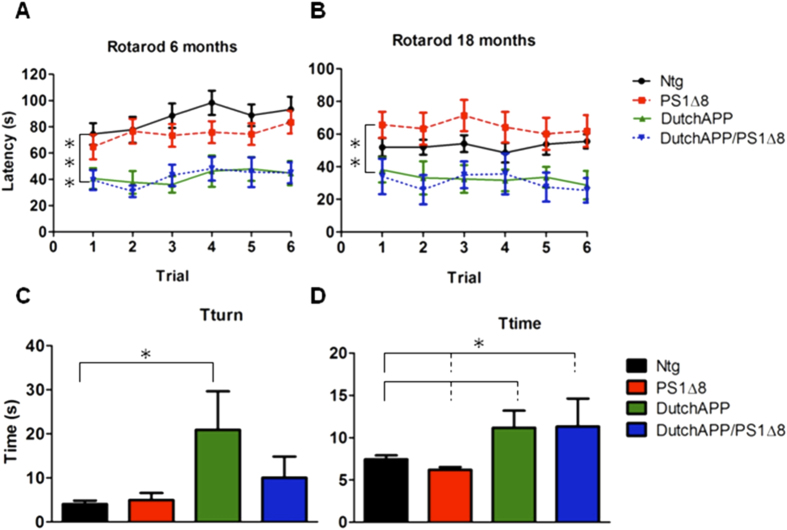
The PS1^∆exon8^ mutation transgene does not exacerbate the motor impairments exhibited by FAD Dutch APP. (**A**) In the rotarod test, there was a significant decrease in average latency to fall in 6 month-old FAD Dutch APP and FAD Dutch APP/PS1^∆exon8^ mice compared to Ntg and PS1^∆exon8^-only mice. (**B**) There was also a significant decrease in average latency to fall in 18 month-old FAD Dutch APP and FAD Dutch APP/PS1^∆exon8^ mice compared to Ntg and PS1^∆exon8^-only mice. (**C**) In the pole test, there was a significant difference between FAD Dutch APP and Ntg in Tturn with FAD Dutch APP mice taking a significantly longer time to turn. (**D**) There was also a significant difference between FAD Dutch APP and FAD Dutch APP/PS1^∆exon8^ in time compared to Ntg and PS1^∆exon8^-only. In none of the behavioral assays utilized did the PS1^∆exon8^ mutation exacerbate behavioral impairments relative to Dutch APP only. Black = Ntg, Red = PS1^∆exon8^, Green = FAD Dutch APP, Blue = FAD Dutch APP/PS1^∆exon8^. N = 29 Ntg, 30 PS1^∆exon8^, 17 FAD Dutch APP, and 12 FAD Dutch APP/PS1^∆exon8^ (6 months) and 23 Ntg, 19 PS1^∆exon8^, 9 FAD Dutch APP, and 7 FAD Dutch APP/PS1^∆exon8^ (18 months). N = 19 Ntg, 16 PS1^∆exon8^, 11 FAD Dutch APP, and 10 FAD Dutch APP/PS1^∆exon8^ (pole test). Values represent means ± SEM, *p < 0.05, **p < 0.01, ***p < 0.001.

It is important to recall that humans harboring *PSEN1*^*∆exon8*^ alleles displayed unusual motor features^9^. Therefore, we sought to determine whether the PS1^∆exon8^ mutation expressed in the presence of two wt mouse PS1 alleles resulted in behavioral deficits similar to the human motor phenotypes observed in patients with the L271V mutation. We found a significant decrease in rotarod latency to fall in 6 and 18 month-old FAD Dutch APP (6 months = 42.17 ± 1.97 s; 18 months = 32.90 ± 1.25 s) and FAD Dutch APP/PS1^Δexon8^ mice (6 months = 41.92 ± 2.51 s; 18 months = 30.56 ± 1.94 s) compared to Ntg (6 months = 86.84 ± 3.71 s; 18 months = 52.63 ± 1.00 s) and PS1^Δexon8^-only mice (6 months = 74.73 ± 2.48 s; 18 months = 64.44 ± 1.59 s; F_(3, 80)_ = 7.727, p = 0.0001, 6-month time-point, F_(3, 50)_ = 5.003, p = 0.004, 18 month time-point; Fig. 3A,B). Of note, there was no significant difference in weight amongst the four genotypes. Mice were also tested on the fear conditioning and basic locomotor tasks, but no discernable differences amongst the genotypes were noted.

To explore further if the presence of the PS1^∆exon8^ mutation affected the motor skills of the FAD Dutch APP mice, we tested 18 month-old mice on the pole test. The Levene’s test of homogeneity was significant, so we ranked the values for each animal (1 scoring the fastest time) until all animals were ranked. The average of the ranks was then taken for each genotype and a one-way ANOVA was performed. There was a significant difference in Tturn (s) between FAD Dutch APP (20.88 ± 8.38) and Ntg (4.07 ± 0.78; F_(3, 52)_ = 3.02, p = 0.034) and in Ttime (s) for FAD Dutch APP (11.17 ± 1.93) and FAD Dutch APP/PS1^∆exon8^ mice (11.30 ± 3.32) compared to Ntg (7.44 ± 0.47) and PS1^∆exon8^ mice (6.19 ± 0.33). The FAD Dutch APP mice took significantly longer to turn and to go down the pole compared to either Ntg or PS1^∆exon8^-only mice (F_(3, 52)_ = 3.18, p = 0.039, Fig. 3C,D). This result is consistent with the interpretation that the PS1^∆exon8^ transcripts produced by PS1 L271V were probably not contributory to the either the behavioral or the motor phenotypes.

### Double-transgenic FAD Dutch APP/PS1^∆exon8^ mice do not show altered Aβ42/40 ratios and do not develop extracellular Aβ plaques

**Figure 4:**
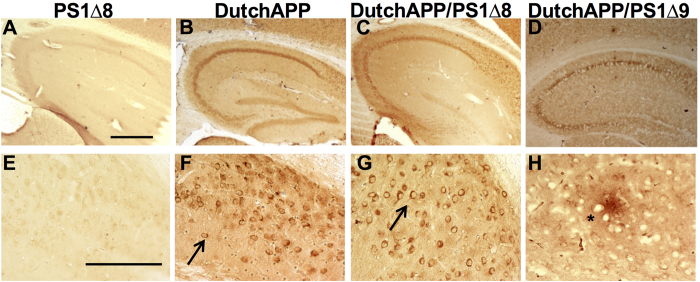
FAD Dutch APP/PS1^∆exon8^ bigenic mice do not display extracellular amyloid plaques. APP species were visualized using the monoclonal 6E10 antibody. (**A**) PS1^∆exon8^ expression on its own does not exhibit human APP immunoreactivity. (**B**) FAD Dutch APP mice (18 months-old) exhibit intracellular APP/Aβ-like immunoreactivity. (**C**) FAD Dutch APP/PS1^∆exon8^ mice (18 months-old) also exhibit intracellular APP/Aβ-like immunoreactivity. (**D**) In contrast, another early-onset AD PS1 mutation, PS1^∆exon9^, when crossed with FAD Dutch APP, does lead to extracellular plaques. (**E**) Higher magnification of PS1^∆exon8^ in (**A**), showing no APP immunoreactivity. (**F**) Higher magnification of the intracellular APP/Aβ-like immunoreactivity of FAD Dutch APP (APP-Aβ-LIR; arrow) in (**B**). (**G**) Higher magnification of FAD Dutch APP/PS1^∆exon8^ in (**C**), showing the intracellular APP/Aβ-like immunoreactivity (APP-Aβ-LIR; arrow). (**H**) Higher magnification of the FAD Dutch APP/PS1^∆exon9^ plaque seen in (asterisk) (**D**) All images are from the hippocampus. Scale, (**A–D**), 2 mm and (**E–H**), 200 μm.

As an additional *in vivo* test in brain of the potential amyloidogenicity and pathogenicity of *PSEN1*^*∆exon8*^ *, w*e employed bigenic mouse models to assess whether PS1^∆exon8^ could promote cerebral amyloidosis as we previously reported when we crossed FAD Dutch APP mice with another line of mice harboring a deletion mutation in the adjacent exon^16^ (i.e., exon 9). Consistent with the results reported using cultured cells^9^, we detected no differences in hippocampal Aβ40 or Aβ42 levels, or the Aβ42/40 ratio when brain extracts from FAD Dutch APP mice were compared with brain extracts from FAD Dutch APP/PS1^∆exon8^ mice (Supplemental Table 1). Transgenic mice expressing mutant PS1 alone never develop Aβ deposition in the brain because mouse Aβ is much less prone to aggregation than human Aβ (Fig. 4A,E, PS1^∆exon8^-only). At 18 months of age, we confirmed the absence of Aβ deposition in the FAD Dutch APP mutant mice (Fig. 4B,F; See also^16^), and in the FAD Dutch APP/PS1^∆exon8^ mutant mice (Fig. 4C,G). However, when we crossed FAD Dutch APP mice with PS1^∆exon9^ mice, we observed plaque deposition in the FAD Dutch APP/PS1^∆exon9^ mice by 11 months of age (Fig. 4D,H; See also^16^). This is consistent with the differential ability of PS1^∆exon8^ vs PS1^∆exon9^ to promote excess Aβ42 generation^9^ and constitutes what we would interpret as definitive evidence that PS1^∆exon8^ is probably not the transcript responsible for cerebral amyloidosis and Tas-1 FAD^9^.

### PS1^∆exon8^ interacts with wt human PS1 in HEK 293T cells

Our earlier studies had implicated the modulation of wt PS1 action by PS1^∆exon8^; yet, PS1-PS1 interactions are not explicitly accountable using the Wolfe 1:1:1:1 model^12^. We used co-immunoprecipitation (co-IP) and bimolecular fluorescence complementation (BiFC) to determine whether PS1^∆exon8^ was involved in a physical interaction with wt PS1.

**Figure 5:**
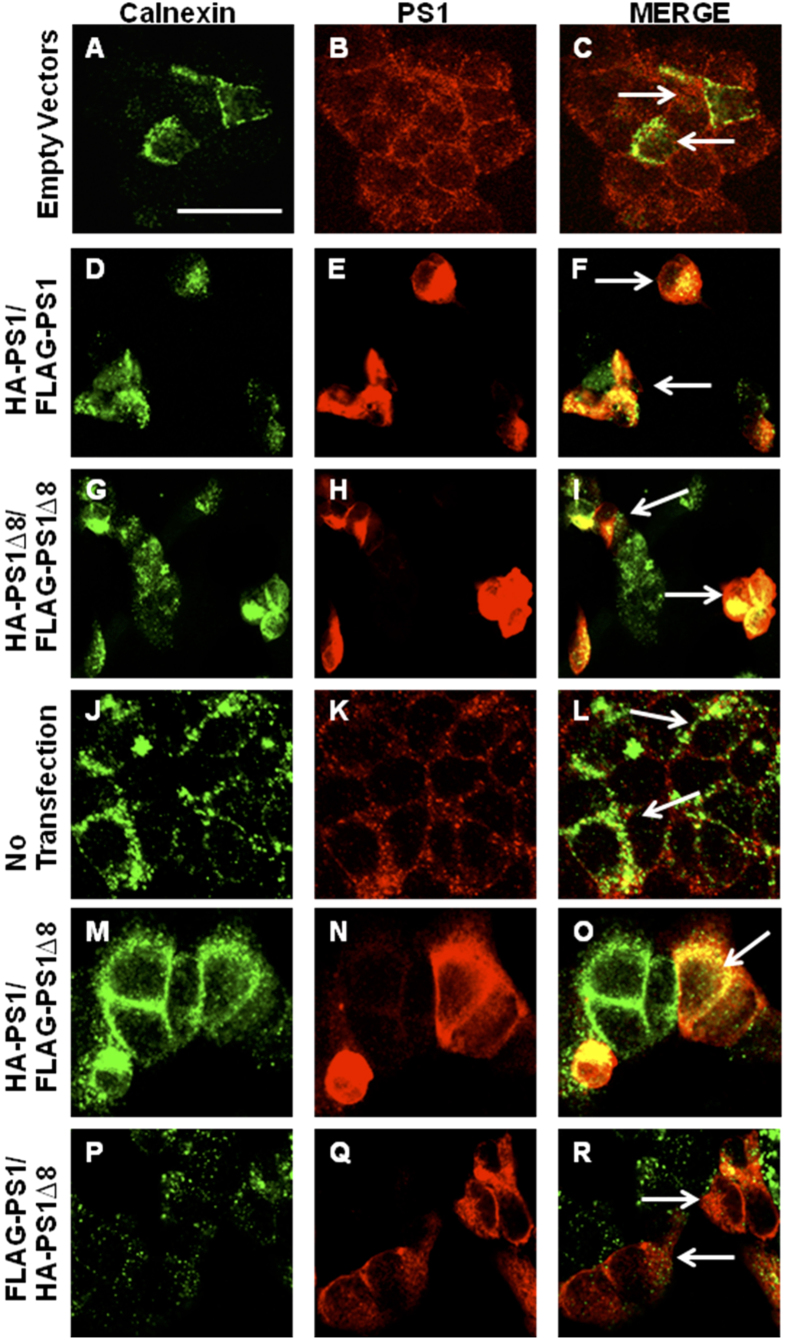
HA- and FLAG-tagged PS1 and PS1^∆exon8^ are localized to the endoplasmic reticulum (ER). HEK 293T cells were transfected with the same construct combinations described in the Results section. After 24 hrs, cells were fixed in 4% paraformaldehyde and immunostained for calnexin (green), a marker for the ER (left panel) and for human PS1 (red, middle panel) showing that the HA- and FLAG-tags do not disrupt normal PS1 colocalization indicated by white arrows (right panel) compared to the Empty Vector and No Transfection conditions which contain endogenous levels of human PS1. (**A–C**) Empty Vectors. (**D–F**) HA-PS1 and FLAG-PS1. (**G–I**) HA-PS1^∆exon8^ and FLAG-PS1^∆exon8^. (**J–L**) No Transfection (**M–O**). HA-PS1 and FLAG-PS1^∆exon8^. (**P**–**R**) FLAG-PS1 and HA-PS1^∆exon8^. Scale bar, 200 μm. See supporting material in Supplemental Fig. 1.

**Figure 6:**
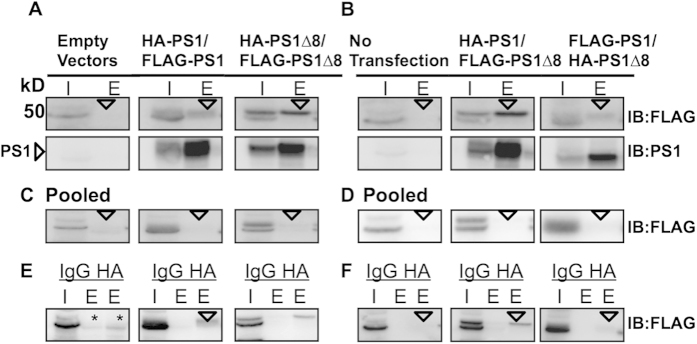
PS1 and PS1^∆exon8^ co-immunoprecipitate *in vitro*. Co-IP immunoblots of different epitope-tagged PS1 and PS1^∆exon8^ proteins. Constructs were cotransfected in six different combinations: (**A**) (1) empty vectors containing either HA or FLAG only, (2) HA-tagged PS1 and FLAG-tagged PS1, and (3) HA-tagged PS1^∆exon8^ and FLAG-tagged PS1^∆exon8^. (**B**) (4) No transfection, (5) HA-tagged PS1 and FLAG-tagged PS1^∆exon8^, and (6) FLAG-tagged PS1 and HA-tagged PS1^∆exon8^. Transfected constructs were co-immunoprecipitated with anti-HA antibody (1.5 μg per 300 μg cell lysate) and blotted with the anti-FLAG antibody (top immunoblot) and the anti-human PS1 antibody NT.1 (directly below FLAG immunoblot). The co-immunoprecipitated band is indicated by arrows and corresponds to full-length PS1 (~50 kDa). Note there is no band present in the eluate lane for Empty Vectors and No Transfection. (**C,D**) For pooled lysates, constructs were transfected in separate wells and then pooled after lysis. The pooled lysed cells were co-immunoprecipitated with anti-HA antibody and blotted for anti-FLAG as described above. No PS1 band was detected in the eluate (indicated by arrows). (**E,F**) The experiment in (**A**,**B**) was repeated but transfected constructs were co-immunoprecipitated with either IgG rabbit as a negative control or the anti-HA antibody. No PS1 band was detected in the IgG control but was detected in the HA lane, except in the Empty Vectors and No Transfection lanes (indicated by arrows, asterisk marks non-specific bands corresponding to the IgG heavy chain). I = input, E = eluate, IgG = rabbit IgG, and HA = HA antibody.

We first established that the HA- and FLAG-tags on PS1 did not alter PS1 subcellular localization. We observed that all the tagged proteins localized to the endoplasmic reticulum (ER) and plasma membrane, where PS1 is normally found in the cell (Fig. 5). To assay for interactions via co-IP, plasmids were co-transfected in six different combinations: (1) HA-tagged PS1 and FLAG-tagged PS1; (2) HA-tagged PS1^∆exon8^ and FLAG-tagged PS1^∆exon8^; (3) HA-tagged PS1 and FLAG-tagged PS1^∆exon8^; (4) FLAG-tagged PS1 and HA-tagged PS1^∆exon8^; (5) empty HA-tagged vector and empty FLAG-tagged vector (neither containing PS1 nor PS1^∆exon8^, as a negative control); and (6) GFP only (as a positive transfection control), in order to determine whether an HA-tagged PS1 or PS1^∆exon8^ protein could co-immunoprecipitate with a FLAG-tagged PS1 or PS1^∆exon8^ protein. Proteins from total cellular extracts were immunoprecipitated with anti-HA antibody (1.5 μg per 300 μg lysate) and then immunoblotted with anti-FLAG antibody, anti-human PS1 NT.1 antibody, and anti-HA antibody. We show that the HA-tagged PS1 and FLAG-tagged PS1 protein and HA-tagged PS1^∆exon8^ and FLAG-tagged PS1^∆exon8^ protein complexes co-immunoprecipitated, providing evidence for physical interaction and the possible formation of homodimers (Fig. 6A). We also found that the HA-tagged PS1 protein co-immunoprecipitated with FLAG-tagged PS1^∆exon8^ protein and that the FLAG-tagged PS1 protein co-immunoprecipitated with HA-tagged PS1^∆exon8^ protein, potentially forming heterodimers (Fig. 6B). To confirm that the interaction between the tagged PS1 and PS1^∆exon8^ proteins was occurring in live cells and was not an artifact of cell lysis, the plasmids were individually transfected in separate tissue culture wells and extracts were pooled after lysis. The pooled, lysed cells were co-immunoprecipitated with 1.5 μg anti-HA antibody per 300 μg lysate and blotted for anti-FLAG as described above. A PS1 band was not detected in the eluate (Fig. 6C,D). A final control experiment was performed to ensure that the interaction between the differently tagged PS1 proteins was specific to the antibody and not to the IgG control. The co-IP experiment was repeated but transfected proteins were co-immunoprecipitated with either rabbit IgG as a negative control or the HA antibody. Again, a PS1 band was not detected in the IgG control (Fig. 6E,F).

**Figure 7:**
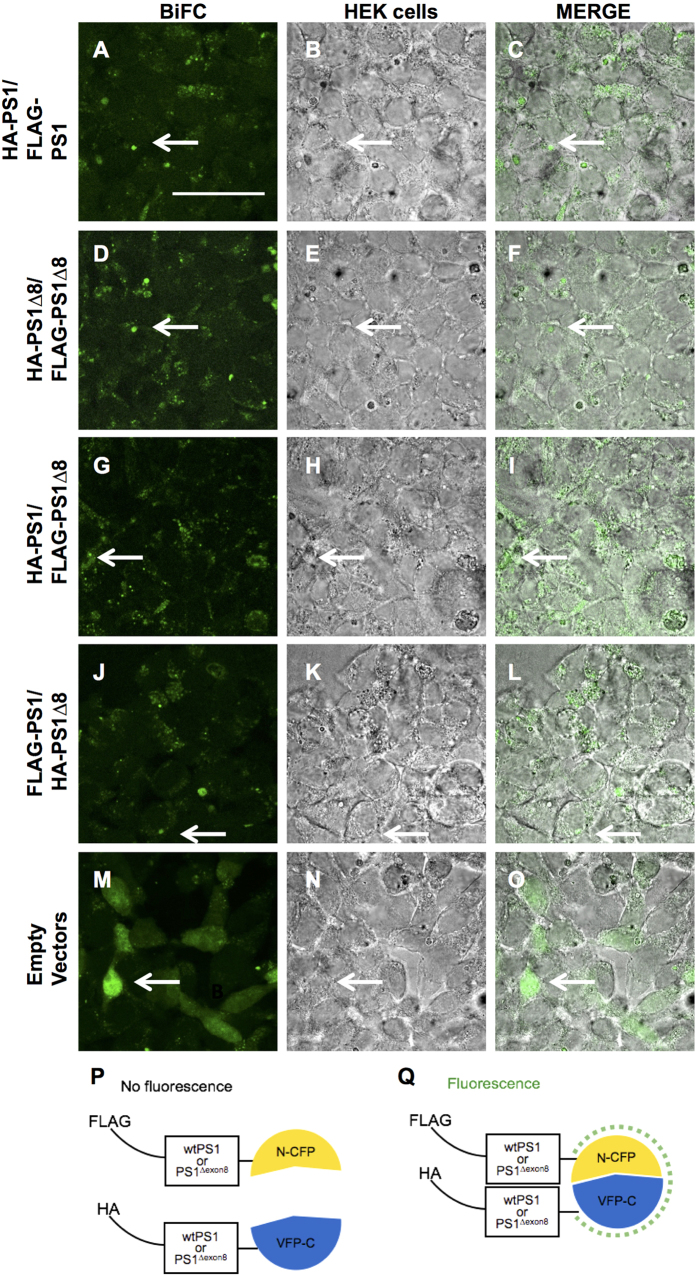
BiFC assay reveals interaction between PS1 and PS1^∆exon8^ proteins. Interaction between PS1-PS1 and PS1^∆exon8^-PS1^∆exon8^ and PS1-PS1^∆exon8^ occurs in vesicles near the plasma membrane (indicated by arrows). Left panel shows the BiFC fluorescence image excited with a 488 nm laser. Middle panel shows HEK 293T cells imaged with brightfield, and the right panel is the overlay. (**A–C**) HA-tagged PS1 CT Venus fluorophore and FLAG-tagged PS1 NT Cerulean fluorophore (**D–F**) HA-tagged PS1^∆exon8^ CT Venus fluorophore and FLAG-tagged PS1^∆exon8^ NT Cerulean fluorophore (**G–I**) HA-tagged PS1 CT Venus fluorophore and FLAG-tagged PS1^∆exon8^ NT Cerulean fluorophore (**J–L**) FLAG-tagged PS1 NT Cerulean fluorophore and HA-tagged PS1^∆exon8^ CT Venus fluorophore. (**M–O**) Empty vector reveals an overall cell-like staining. Scale bar, 200 μm. (**P,Q**) Schematics for vector preparations and reporting fluorescence interactions.

We employed BiFC to visualize the PS1 and PS1^∆exon8^ protein-protein interaction in whole cells. The plasmid combinations described above were transfected in 8-well chamber slides. An interaction was detected immunocytochemically following transfection of all four combinations: HA-tagged PS1 and FLAG-tagged PS1 (Fig. 7A–C), HA-tagged PS1^∆exon8^ and FLAG-tagged PS1^∆exon8^ (Fig. 7D–F), HA-tagged PS1 and FLAG-tagged PS1^∆exon8^ (Fig. 7G–I), and FLAG-tagged PS1 and HA-tagged PS1^∆exon8^ (Fig. 7J–L). The interaction appears to be occurring in vesicles that display a distinctly different distribution from that observed using empty vectors, in which the fluorescence is noted to be filling the entire cytoplasm (Fig. 7M–O).

### PS1^∆exon8^ interacts with nicastrin in PS1^∆exon8^ transgenic mouse brain

**Figure 8:**
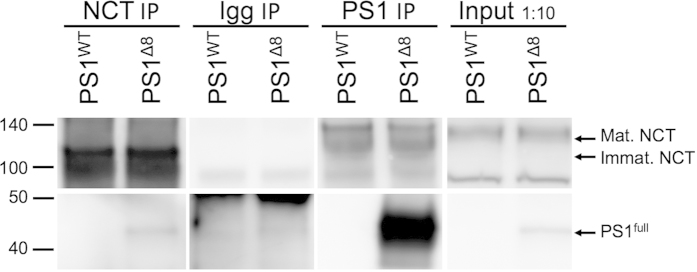
Nicastrin and PS1^∆exon8^ co-immunoprecipitate *in vivo*. Co-IP immunoblots of forebrain lysates of PS1^∆exon8^-positive mice and controls. Top panel contains blots for nicastrin (NCT) made with an antibody targeting the C-terminus of human nicastrin (amino acids 693–709). Bottom panels are blotted with a monoclonal anti-human PS1 specific antibody that detects full length PS1. Columns from left-to-right include immunoprecipitated lysates using a NCT antibody (N1660; Sigma), IgG negative control (sheep anti-rabbit M-280 Dynabeads), PS1 antibody for positive control (D3931; Cell Signaling) (See also Supplemental Fig. 2), and diluted input 1:10. Note that full–length PS1 bands were only detected in PS1^∆exon8^ lysates (~45 kDa), except in the IgG negative control. NCT immunoprecipitation detected mostly immature forms of NCT (~115 kDa), as compared to PS1 positive controls, and inputs. NCT immunoprecipitation picked up immunoblots in PS1^∆exon8^ lanes, corresponding to full-length PS1 indicating that these two proteins interact.

As noted above, our earlier studies had implicated the modulation of wt PS1 action by PS1^∆exon8^ apparently violating the Wolfe 1:1:1:1 model^12^. As also described above, we used BiFC to demonstrate that there exists a physical basis for the modulation of wt PS1 action by PS1^∆exon8^. Having demonstrated this wt PS1: PS1^∆exon8^ interaction, we were also interested to determine whether PS1^∆exon8^ integrates into γ-secretase complexes *in vivo* or whether this inactive species was excluded from these complexes . Specifically, using co-IP, we tested whether PS1^∆exon8^ interacts with nicastrin, the first step in formation of the γ-secretase complex. For these experiments, we focused on full length PS1, since its detection is mainly present in PS1^∆exon8^ as compared to wt PS1^9^ (Fig. 8 right panel). Using an anti-nicastrin antibody for immunoprecipitation, followed by Western blotting for components of the γ-secretase complex, we were able to detect the presence of full-length PS1. This result supports the hypothesis that PS1^∆exon8^ forms physical complexes with nicastrin and is competent to be incorporated into γ-secretase complexes. Anti-nicastrin immunoprecipitation lysates showed recovery of a full-length PS1 species that was present in the protein extracts from PS1^∆exon8^ positive mice, but not in extracts from wt mice (Fig. 8 left panel). The PS1^∆exon8^ : nicastrin interaction is likely to involve immature nicastrin since this is typically the predominant form of nicastrin pulled down in standard γ-secretase complex immunoprecipitation experiments^8^,^12^.

## Discussion

In the current study, our specific goals were to determine whether PS1^∆exon8^ could rescue the lethal Notch-deficient phenotype^14^,^15^ of the PS1-null mouse and to determine whether PS1^∆exon8^ could form complexes with wt PS1 and/or nicastrin. To this end, we demonstrated herein that PS1^∆exon8^ cannot rescue the embryonic lethality phenotype of the PS1 KO *in vivo*, and that PS1^∆exon8^ physically interacts with wt PS1 molecules in culture and with nicastrin in brain (see Figs 6, 7, 8).

A single copy of the PS1^∆exon8^ transgene introduced into wt mice resulted in only a mild behavioral phenotype, perhaps because the two copies of endogenous mouse PS1 compensate for the presence of PS1^∆exon8^. We also crossed PS1^∆exon8^ with the previously described FAD Dutch APP mice^16^, and we found that double heterozygous mice (i.e., FAD Dutch APP +/− X PS1^∆exon8^ +/− mice) displayed no exacerbation of the behavioral deficits, or the vascular and parenchymal amyloid pathology in the FAD Dutch APP mice. In contrast, FAD Dutch APP +/− X PS1^∆exon9^ +/− double heterozygous mice lacking the adjacent exon (i.e., exon 9 is deleted rather than exon 8) show robust amyloid pathology in both the cerebral vasculature and the parenchyma. From a structure-activity relationship perspective, it is worth noting that the failure of PS1^∆exon8^ to exacerbate behavioral deficits or histopathology distinguishes the molecular consequences of PS1^∆exon8^ from those associated with deletion of the adjacent exon (exon 9)^9^,^17^.

The phenotype associated with the concurrent expression of PS1^∆exon8^ and PS1 wt alleles is also different from that associated with either the PS1/2 DKO state or the concurrent presence of one PS1 wt allele and one PS1 allele containing single or double mutations of active site aspartate(s) to alanine(s). In these latter two situations, the aspartate mutants act as dominant negatives so that γ-secretase activity becomes undetectable^18^. In contrast, when PS1^∆exon8^ and PS1 wt are coexpressed, there is a compound phenotype that includes both quantitatively reduced catalysis of APP β-carboxyl terminal fragments to form Aβ and qualitatively abnormal γ-secretase function so that a range of aberrant Aβ peptide species is generated^9^. PS1^∆exon8^ is therefore associated with both *hypo*function of γ-secretase and qualitative *dys*function of γ-secretase complexes occurring *in trans.* We cannot, at this point, determine whether the PS1^∆exon8^ that interacts with the wt molecules has been incorporated into separate complexes or whether non-complexed PS1^∆exon8^ interacts with a normally complexed and fully active wt counterpart.

Other investigators have tested the complex-competence of pathogenic FAD mutations in PS1, and, to date, all FAD pathogenic mutant PS1 molecules are incorporated into γ-secretase complexes^19^,^20^. Of note, this also applies to PS1^∆exon9^, which, although different from PS1^∆exon8^ in important aspects as discussed above, is similar to PS1^∆exon8^ in causing an exon deletion and is associated with both hypofunction of γ-secretase and qualitative dysfunction of γ-secretase^9^,^17^.

With regard to this loss of catalytic function, it is worth noting that aspartate mutant PS1 molecules that lack catalytic function and act as dominant-negative modulators represent the only currently known example whereby a mutant PS1 is not properly incorporated into γ-secretase complexes^21^. One might speculate, therefore, that loss of function might be associated with failure to form γ-secretase complexes. However, PS1 L166P and C410Y are also dramatically hypofunctional, producing Aβ at only about 10% of the levels generated by wt PS1, yet they are apparently incorporated into γ-secretase complexes normally^4^. The PS1^∆exon8^ molecule appears to exhibit similar behavior and both protein-protein interactions and cellular localization consistent with successful packaging into γ-secretase complexes (Figs 5, 6, 7, 8).

The 1:1:1:1 model of γ-secretase complex stoichiometry notwithstanding^12^, two additional independent reports provide support for our suggestion that mutant PS1 interacts *in trans* with wt PS1. One of these^2^ describes a loss-of-function mutant PS1, L435F, which, when expressed with APP-C99 in PS1/2 DKO mouse embryonic fibroblasts resulted in almost no rescue of Aβ40 and Aβ42 production. However, when coexpressed with wt PS1, PS1 L435F compromised the ability of wt PS1 to produce Aβ40, as indicated by reduced total Aβ40 production that fell below the level produced by wt PS1 alone. This is quite similar to the effect of PS1^∆exon8^. However, co-expression of the L435F PS1 mutant with wt PS1 resulted in Aβ42 levels greater than wt PS1 alone^13^, and this Aβ42-elevating property is not applicable to PS1^∆exon8^ 9. With regard to γ-secretase complex formation, Heilig *et al.*^13^ demonstrated that alternately FLAG- or HA-tagged wt or L435F PS1 mutant molecules could be recovered by co-IP with either anti-FLAG or anti-HA. We provide comparable evidence to support the formation of complexes involving wt PS1 and PS1^∆exon8^.

In summary, we have demonstrated that, by all standard criteria, a physiologically generated alternative PS1 transcript lacking exon 8 lacks catalytic activity in brain *in vivo*, as predicted from studies in cell culture^9^. Despite current concepts about the structure and stoichiometry of functional γ-secretase complexes^12^, PS1 molecules with complete (this study) or dramatic^2^,^13^ loss of catalytic function can interact with wt PS1 molecules *in trans* and remain competent for at least the initial step (i.e., formation of complexes with nicastrin) toward biogenesis of γ-secretase complexes.

## Methods

### Animals

APP^E693Q^ (Dutch APP), double transgenic Dutch APP/PS1^∆exon9^, PS1^∆exon8^, and double transgenic Dutch APP/PS1^∆exon8^ mice were used in the current study. Generation of the FAD Dutch APP and FAD Dutch APP/PS1^∆exon9^ transgenic mouse lines was previously described^16^. The PS1^∆exon8^ cDNA was excised from its parental plasmid and inserted into the pENTR4 plasmid (Life Technologies, Carlsbad, CA) at the KpnI and NotI sites. The PS1^∆exon8^ cDNA was then excised from pENTR4 with SalI and XhoI and inserted into the Thy-1 plasmid at the unique XhoI site. To make the PS1^∆exon8^ transgenic mouse, the DNA was linearized with PvuI, purified from an agarose gel, and dialyzed before standard pronuclear injection. All mice were backcrossed onto a C57Bl6/J background.

To generate transgenic human PS1^∆exon8^(+) (hPS1^∆exon8^) mice on a mouse PS1-null (*−/−*) background, heterozygous PS1^∆exon8^(+/−) mice were crossed with heterozygous mice PS1(+/−) (mPS1) described in reference^15^. Mice with the genotype hPS1^∆exon8^(−)/mPS1(+/−) and hPS1^∆exon8^(+)/mPS1(+/−) were crossed in order to obtain embryos of genotypes hPS1^∆exon8^(+)/mPS1(+/−), hPS1^∆exon8^(+)/mPS1(*−/−*), hPS1^∆exon8^(+)/mPS1(+/+), hPS1^∆exon8^(−)/mPS1(+/−), hPS1^∆exon8^(−)/mPS1(*−/−*), and hPS1^∆exon8^(−)/mPS1(+/+). For all mice, tails were biopsied and DNA extracted for transgene analysis. Mice were genotyped for the presence of the PS1^∆exon8^ or PS1^∆exon9^ transgene (forward 5′- CCCATTCACAGAAGATACCGAGAC-3′; reverse 5′-CGTGGCTCATCTTGGCTGTGAT-3′), Dutch APP (E693Q) transgene (forward 5′-CCGATGATGACGAGGACGAT-3′; reverse: 5′-TGAACACGTGACGACGCCGA-3′), and the mouse PS1 gene^15^.

All mice used were group housed under a 12-hr light/dark cycle and given *ad libitum* access to food and water. All animal procedures were conducted in accordance with the National Institute of Health Guidelines for the Care and Use of Experimental Animals and were approved by the Institutional Animal Care and Use Committee at the Icahn School of Medicine at Mount Sinai.

### Generation of plasmids

Wildtype human PS1 and PS1^∆exon8^ were subcloned via PCR into two different bimolecular fluorescence complementation (BiFC) vectors (gifts from Dr. Chang-Deng Hu, Purdue University): the VC155 construct was tagged with an HA-tag at the N-terminus and a Venus fluorescent protein at the C-terminus (BiFC construct VC155; sequence available on Addgene at www.addgene.org) while the CrN173 construct contained a FLAG-tag at the N-terminus and a Cerulean fluorescent protein at the C-terminus (BiFC construct CrN173; sequence available on Addgene). After PCR amplification, PS1 and PS1^∆exon8^ were digested with EcoRI and SalI and inserted into the HA-tagged vector directly 3′ of the HA sequence [VC155 (HA-tagged vector)] in the EcoRI and XhoI restriction sites. FLAG-tagged PS1 and PS1∆8 vectors were synthesized by GENEWIZ (South Plainfield, NJ). PS1 and PS1^∆exon8^ were digested with HindIII and XbaI and inserted into the FLAG-tagged vector directly 3′ to the FLAG sequence [CrN173 (FLAG-tagged vector)]. Plasmid nucleotide sequences were confirmed by DNA sequencing (GENEWIZ).

### RNA analysis

Embryo brains were removed from the skull, and the left hemisphere was snap-frozen for Western blot analysis while the right hemisphere was snap-frozen for RNA extraction. Total RNA was extracted using the Ambion RNA extraction kit (Life Technologies, Carlsbad, CA). One μg of RNA was reverse-transcribed using the Superscript II RT enzyme (Invitrogen, Life Technologies) and a poly(dT) primer (Invitrogen, Carlsbad, CA) followed by PCR amplification using the primers (forward 5′-CCCATTCACAGAAGATACCGAGAC-3′; reverse

5′-GAGTCACAAGACACTGTTGCAGAG-3′) flanking the PS1∆8 deletion.

### Western blotting

The embryonic brain was rapidly dissected, and the left hemisphere was dissociated by 20 strokes of a glass-Teflon homogenizer at 500 rpm in 1% Triton X-100/TBS (pH 7.6) with protease/phosphatase inhibitors [1 mM EDTA, 1 mM Na_3_VO_4_, 5 μM ZnCl_2_, 100 mM NaF, 1 μM pepstatin, 1 mM PMSF, mini-complete protease inhibitor tablet (Roche, Indianapolis, IN)], followed by centrifugation at 10000 × g for 20 minutes. Protein concentrations were determined using the Pierce BCA Protein Assay Kit (Thermo Scientific, Waltham, MA). Protein lysates were diluted to 1× in sample buffer (20 mM Tris, 1% glycerol, 180 mM β-mercaptoethanol, 0.003% bromophenol blue, and 2% SDS, pH 6.8) and heated at 70° for 10 minutes before being loaded onto 4–12% Bis-Tris SDS page gels (Biorad, Hercules, CA) for electrophoresis with NuPage 1× MES/SDS running buffer (Invitrogen, Grand Island, NY). The gels were electrophoretically transferred onto a 0.2-μm PVDF membrane (Millipore, Billerica, MA) and blotted with primary antibody as indicated (anti-human PS1 mAb NT.1; 1:500 dilution; Millipore and monoclonal anti-β-actin; 1:1000 dilution; Sigma, St. Louis, MO) overnight at 4 °C in 5% w/v non-fat milk (Santa Cruz Biotechnology, Santa Cruz, CA) in TBS containing 0.1% v/v Tween-20 (Fisher Scientific, Pittsburgh, PA; TBS-T). The blots were then washed 6 × 15 minutes before being visualized using an HRP-conjugated goat anti-rat secondary antibody (1:5000 dilution for anti-human PS1 mAb NT.1; Santa Cruz Biotechnology) and HRP-conjugated goat anti-mouse IgG (Fab-specific) secondary antibody (1:20,000 dilution for the monoclonal anti-β-actin antibody; Sigma). Membranes were washed once in TBS-T and stripped in low pH stripping buffer [25 mM glycine HCl, pH 2.0 and 1% w/v SDS] with vigorous shaking to remove primary and secondary antibody, washed 3× in TBS-T, and blocked for 1 hr (in 5% milk/TBS-T) at room temperature before probing with the next primary antibody. Signals were detected by enhanced chemiluminescence (Pierce, Thermo Scientific) and digital images were captured using LAS3000 (Fujifilm, Tokyo, Japan).

### Hematoxylin-eosin histochemistry

Embryos were placed in a 4% paraformaldehyde (PFA) solution followed by equilibration to a 30% sucrose solution before being cut into 20 μm-thick sections on a cryostat (Leica Biosystems, Wetzlar, Germany). Sections were pretreated in a series of xylene and ethanol gradient followed by hematoxylin (Thermo Scientific) and eosin (Fisher Scientific) treatment for three minutes each. The tissue then went through another series of dehydration steps and was cover-slipped with Cytoseal (Thermo Scientific). Images were captured using a 10×/0.32 N.A. Plan-Apochromat objective and an oil immersion 40×/1.3 N.A. Plan-Apochromat objective on a Zeiss Axiophot microscope (Zeiss, Thornwood, NY).

### Motor behavioral testing

Because spastic paraparesis is clinically associated with the Tas-1 mutation, we tested the motor system in these mice using the rotarod^22^ and pole^23^ tests.

For the rotarod test, 29 non-transgenic littermates (Ntg) (17 males; 12 females), 30 PS1^∆exon8^ (19 males; 11 females), 17 FAD Dutch APP (11 males; 6 females), and 12 FAD Dutch APP/PS1^∆exon8^ (9 males; 3 females) mice at 6 months of age were tested and 23 Ntg (9 males; 14 females), 19 PS1^∆exon8^ (12 males; 7 females), 9 FAD Dutch APP (5 males; 4 females), and 7 FAD Dutch APP/PS1^∆exon8^ (4 males; 3 females) mice for the 18-month time-point were tested. Most, but not all, mice were tested at both 6 and 18 months. For the pole test, there were 19 Ntg (12 males; 7 females), 16 PS1^∆exon8^ (5 males; 11 females), 11 FAD Dutch APP (9 males; 2 females), and 10 FAD Dutch APP/PS1^∆exon8^ (5 males; 5 females) mice.

Briefly, the rotarod motor task involves placing the mouse on a horizontal rotating cylinder, which gradually accelerates in speed (Columbus Instruments, Columbus, OH). The starting speed for all animals was 8 rpm. The length of time the animal stays on the rotating cylinder is a measure of balance and coordination, physical condition, and motor planning. Each animal was trained for two days, 3 trials each. The third and fourth days (6 total trials) were the test trials. Animals were tested at two time-points: 6 and 18 months. For the 18-month time-point, animals received only one day of training.

The pole test was performed as described^23^. The animals were placed on a 50-cm tall, 8-mm wide wooden rod covered with gauze in order for mice to grip. Animals were placed face up on the pole and were given two days of training, three trials each day, to turn and climb down the pole into a cage. Day 3 was the test day. The time it took the animal to turn (Tturn) and to travel down the pole (Ttime) were recorded and averaged. A total average was calculated for each genotype.

Both males and females were included in the analyses and were not statistically different from each other at 6 months on the rotarod motor task or in T-turn or T-time of the pole test. However, at the 18-month time-point females had a longer latency to fall compared to males (females = 55.89 ± 5.33 s; males = 36.42 ± 4.79 s; F_(3,50)_ = 7.4, p = 0.009), but because of the low N for the 18-month time-point, both males and females were still included.

### Immunohistochemistry

18 month-old (9 wt, 12 PS1^∆exon8^, 9 FAD Dutch APP, and 10 FAD Dutch APP/PS1^∆exon8^, of either sex) mice were perfused transcardially with ice-cold 1% PFA in phosphate buffer followed by 4% PFA as described^24^. Brains were dissected and sectioned on a vibratome (Leica) at 40 μm; every 5^th^ section from a random start was stained using the monoclonal antibody 6E10 [1:1000 anti-Aβ_1–16_, Covance, Princeton, NJ^12^]. Images were captured using a 2.5×/0.075 N.A. and an oil- immersion 40×/1.3 N.A. Plan-Apochromat objectives on a Zeiss Axiophot microscope.

### Co-immunoprecipitation

For co-IP experiments, human embryonic kidney (HEK) 293T cells were cultured at 37 °C/5% CO_2_ in complete growth media (DMEM, 10% FBS and 1% penicillin/streptomycin) and plated in 24-well plates coated with polyornithine. HEK 293T cells were transfected using LipoD293 transfection reagent (SignaGen Laboratories, Rockville, MD) according to the manufacturer’s instructions. Cells were transfected and lysed 24 hrs later (50 mM Tris, pH 7.6, 15 mM NaCl, 1 mM EDTA, 1% NP40, 0.1% SDS, 0.5% sodium deoxycholate, 1 mM PMSF, 1 mM Na_3_VO_4_, 1 mM NaF, complete protease inhibitor cocktail; Roche) and centrifuged at 10,000 g for 20 minutes. Protein concentration was determined using the Pierce BCA Protein Assay Kit (Thermo Scientific). Cell lysates (300 μg) were pre-cleared with prewashed Millipore protein G magnetic beads and rotated at 4 °C for 2 hrs. Beads were removed, and the protein mixture was rotated at 4 °C overnight with either anti-HA antibody (1.5 μg antibody per 300 μg lysate, Sigma) or anti-FLAG antibody (1.5 μg antibody per 300 μg lysate, Sigma). Following overnight incubation, the antibody/protein mixture was rotated at 4 °C for 2 hrs with a fresh 20 μl bead slurry, washed five times, and eluted with 30 μl 4× sample buffer (250 mM Tris, 10% glycerol, 8% SDS, pH 6.8) plus DTT and denatured at 70 °C for 10 min. For each transfection combination, the input, elution, flow-through, and washes were loaded onto a 4–12% Bis-Tris SDS page gel (Biorad) for electrophoresis with NuPage 1× MES running buffer (Invitrogen). The gels were electrophoretically transferred onto a 0.2-μm PVDF membrane and blotted for monoclonal anti-FLAG antibody (1:1000 dilution; Sigma), monoclonal anti-human PS1 NT.1 specific antibody (1:500 dilution; Millipore), and polyclonal anti-HA antibody (1:500 dilution; Sigma) overnight at 4 °C in 5% w/v non-fat milk (Santa Cruz Biotechnology) in TBS containing 0.1% v/v Tween-20 (Fisher Scientific; TBS-T). The blots were then washed 6 × 15 minutes before being visualized using the appropriate HRP-conjugated secondary antibody (Vector Laboratories). Signals were detected by enhanced chemiluminescence (Thermo Scientific) and digital images were captured using LAS3000 (Fujifilm). As a control for the specificity of the co-IP, separate wells were transfected with only one construct then pooled together after lysis^4^. To check for antibody specificity, the co-IP was repeated with each transfection combination being immunoprecipitated with both the HA antibody and a control rabbit IgG.

### Immunocytochemistry and bimolecular fluorescence complementation assay

HEK 293T cells were transfected in 8-well chambered slides (Millipore) coated with polyornithine. At 24 hr post-transfection, cells were washed twice with 1× PBS and fixed for 20 minutes with 4% PFA. Fixed cells were washed with 1× TBS four times, blocked with 1× TBS 3% goat serum/0.25% Triton-X 100 for 30 minutes, then incubated at 4 °C overnight with a polyclonal anti-calnexin antibody (1:1000 dilution; Cell Signaling Technologies, Beverly, MA) and a monoclonal anti-human PS1 specific antibody (1:1000 dilution, provided by Dr. Paul Mathews, NYU), or anti-FLAG (1:1000, Sigma). After overnight incubation, fixed cells were washed in 1× TBS 1% goat serum, incubated with secondary antibody (1:500 dilution; Alexa 488 goat anti-rabbit, Alexa 488 goat anti-mouse and Alexa 594 goat anti-mouse; Invitrogen) for 1 hr at RT, and then washed with 1× Tris buffer before mounting in hard set Vectashield without DAPI (Vector Laboratories). For the BiFC analysis, the experiment was repeated in 4 iterations and at least two regions of interest per iteration were captured. For immunocytochemistry, the experiment was repeated in 3 iterations, and 3 regions of interest per iteration were imaged. Both BiFC and immunocytochemistry experiments were captured on a Zeiss 510 confocal laser-scanning microscope using a 40×/1.4 N.A. Plan-Apochromat objective.

### Co-immunoprecipitation with nicastrin

For *in vivo* co-IP, forebrains from PS1^∆exon8^ positive and wt mice were dissected and homogenized in ice-cold lysing buffer (10 mM HEPES, 150 mM NaCl, 2 mM CaCl_2_, 0.1% Triton X, complete protease inhibitor cocktail; Roche). Protein was measured as described above, and equivalent protein lysates were then precleared with prewashed sheep anti-rabbit M-280 Dynabeads (Life Technologies) (2 hrs, 4 °C). Pre-cleared lysates were then incubated overnight at 4 °C with an anti-nicastrin antibody (4 μl, N1660; Sigma), a PS1 antibody for positive control (4 μl D39D1; Cell Signaling), or without antibody for negative control. After overnight incubation, pre-washed sheep anti-rabbit M-280 Dynabeads were added and allowed to incubate for 6 hours at 4 °C. Beads were then washed 3 times in ice-cold wash buffer (PBS, 0.1% bovine serum albumin, 2 mM EDTA, pH 7.4), and eluted in sample buffer for 15 minutes at 37 °C (20 mM Tris, 1% glycerol, 180 mM β-mercaptoethanol, 0.003% bromophenol blue, and 2% SDS, pH 6.8). Eluted proteins were run on 12% Bis-Tris SDS page gels (Biorad), transferred, and blocked as described above. Gels were then blotted with primary antibody for human anti-nicastrin C-terminus (1:2000, N1660; Sigma), and anti-human PS1 mAb NT.1 (1:2000) overnight at 4 °C in 1% w/v non-fat milk (Santa Cruz Biotechnology, Santa Cruz, CA) in TBS containing 0.1% v/v Tween-20 (Fisher Scientific, Pittsburgh, PA; TBS-T). The blots were then washed 6 × 15 minutes before being visualized with appropriate HRP-conjugated secondary antibody as described above.

### Serial differential detergent fractionation with ultracentrifugation

Mice were exposed to CO2 and perfused transcardially with ice-cold PBS, pH 7.4. Brains were removed and hippocampus and neocortex were dissected, snap-frozen, and stored at −80 °C for biochemical analysis^25^. Briefly, tissue was dissociated by 20 up-and-down strokes of a glass-Teflon homogenizer at 500 rpm at 150 mg/ml (tissue weight) in 1% Triton X-100/TBS (pH 7.6) with protease/phosphatase inhibitors [1 mM EDTA, 1 mM Na_3_VO_4_, 5 μM ZnCl_2_, 100 mM NaF, 1 μM pepstatin, 1 mM PMSF, minicomplete protease inhibitor tablet (Roche, Indianapolis, IN)]. The 1% Triton X-100 homogenate was then ultra-centrifuged at 100,000 × g for 1 hr at 4 °C and the supernatant was removed, aliquoted, and stored at −80 °C until analysis. The 1% Triton X-100-insoluble pellet was then homogenized in 70% formic acid by 20 up-and-down strokes of a glass-Teflon homogenizer at 500 rpm and ultra centrifuged as described above and the liquid phase was removed, neutralized by 1:20 dilution into 1 M Tris (pH 11.0), aliquoted, and stored at −80 °C until analysis.

### Aβ ELISA

Hippocampal and neocortical soluble (1% Triton X-100 fraction) and insoluble (70% formic acid fraction) samples were diluted into ELISA capture buffer to within linear range and were analyzed for concentration of human and mouse Aβ42 and Aβ40 by sandwich ELISA according to the manufacturer’s protocol (Wako, Richmond, VA) (N = 10 Dutch APP mice of either sex and 11 Dutch APP/PS1^∆exon8^ of either sex). Aβ42 and Aβ40 concentrations were corrected for dilution and protein concentration prior to analysis. Total Aβ levels were calculated and concentrations were expressed in pmol/l. Aβ42/40 ratios were also calculated.

### Statistical analyses

All statistical analyses were performed using SPSS v19. For the rotarod motor task, latencies measured in seconds (s) for the last six trials were averaged for each genotype (Ntg, PS1^∆exon8^, FAD Dutch APP, and FAD Dutch APP/PS1^∆exon8^) and repeated measures ANOVA with genotype as the independent variable and latency as the dependent variable was conducted. For the pole test, a one-way ANOVA with genotype as the independent variable and Tturn or Ttime as the dependent variable was performed. Independent samples *t*-tests were used for comparison of FAD Dutch APP to FAD Dutch APP/PS1^∆exon8^. Results are expressed as means ± standard error of the mean (SEM). Statistical significance was set at α = 0.05. A Tukey HSD test was used for post-hoc comparison for differences amongst genotypes, corrected for multiple comparisons. In all instances, a Levene’s test for homogeneity of variance was used for inclusion in parametric tests (p > 0.05 for Levene’s tests).

## Additional Information

**How to cite this article**: Brautigam, H. *et al.* Physiologically generated presenilin 1 lacking exon 8 fails to rescue brain *PS1*–/– phenotype and forms complexes with wildtype PS1 and nicastrin. *Sci. Rep.* **5**, 17042; doi: 10.1038/srep17042 (2015).

## Supplementary Material

Supplementary Information
