# Supplementary material for: Physiologically generated presenilin 1 lacking exon 8 fails to rescue brain PS1−/− phenotype and forms complexes with wildtype PS1 and nicastrin
Source: Sci Rep. 2015 Nov 26;5:17042. doi: 10.1038/srep17042 (PMC4660297; doi:10.1038/srep17042)
Supplement: Supplementary Information [file srep17042-s1.pdf]

Supplementary Information:

Manuscript Title: Physiologically generated presenilin 1 lacking exon 8 fails to rescue brain *PS1-/-* phenotype and forms complexes with wildtype PS1 and nicastrin

Authors: Hannah Brautigam, Cesar L. Moreno, John W. Steele, Alexey Bogush, Dara L. Dickstein, John B.J. Kwok, Peter R. Schofield, Gopal Thinakaran, Paul M. Mathews, Patrick R. Hof, Sam Gandy, and Michelle E. Ehrlich.

ELISA Hippocampus			
	**Aβ40** pmol/l	**Aβ42** pmol/l	**Aβ42/40** x100
**Dutch APP/ PS1Δ8**	**107.4 ± 17.8**	**5.6 ± 1.2**	**9.7 ± 3.1**
**Dutch APP**	**106.9 ± 16.9**	**5.4 ± 0.7**	**7.2 ± 1.8**

ELISA Neocortex			
	**Aβ40** pmol/l	**Aβ42** pmol/l	**Aβ42/40** x100
**Dutch APP/ PS1Δ8**	**76.9 ± 5.6**	**9.3 ± 1.6**	**13.3 ± 3.3**
**Dutch APP**	**109.4 ± 7.4**	**9.6 ± 1.4**	**9.1 ± 1.3**

Supplemental Table 1. Dutch APP/PS1∆8 mice exhibit no change in human/mouse soluble or insoluble Aβ in the hippocampus or neocortex compared to Dutch APP>only mice. Dutch APP/PS1∆8 mice (N = 11) show no change in total Aβ40, Aβ42, or the Aβ42/40 ratio in the hippocampus or neocortex brain regions compared to Dutch APP-only mice (N = 10). Total = sum of soluble (Triton-X fraction) and insoluble (Formic acid) fraction. Mean values ± SEM measured in pmol/l. *t*-test comparisons were performed for Aβ40, Aβ42, or the Aβ42/40 ratio in the hippocampus or neocortex between Dutch APP/PS1∆8 mice and Dutch APP-only.

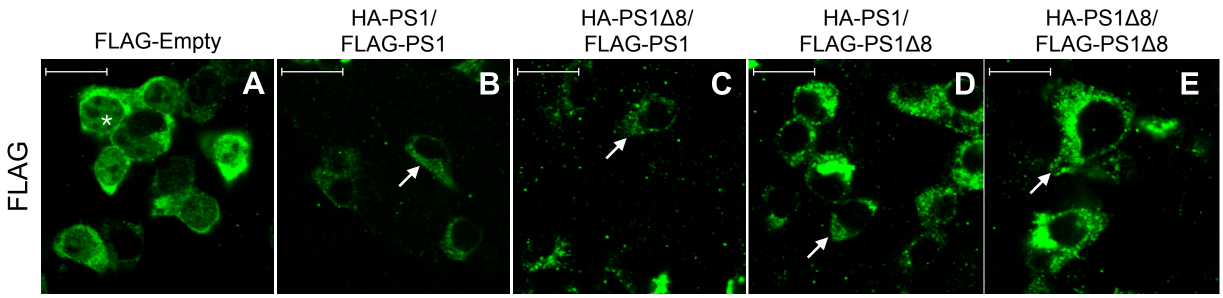

Supplemental Figure 1. HA- and FLAG-tagged PS1 and PS1$^{\Delta exon8}$ are localized to the endoplasmic reticulum (ER). HEK 293T cells were transfected with the same construct combinations described in the Results section. After 24 hrs, cells were fixed in 4% paraformaldehyde and immunostained for Anti-FLAG (green). HA- and FLAG-tags do not disrupt normal PS1 colocalization (restrained to cell membrane and possible ER) as indicated by white arrows (**B-E**), compared to the Empty Vector which contains ubiquitous expression of the FLAG tag indicated by asterisk (includes more intracellular structures) (**A**). Anti-FLAG staining. **A** Empty Vectors. **B** HA-PS1 and FLAG-PS1. **C**. HA-PS1$^{\Delta exon8}$ and FLAG-PS1. **D**. HA-PS1 and FLAG-PS1$^{\Delta exon8}$. **E**. HA-PS1$^{\Delta exon8}$ and FLAG-PS1$^{\Delta exon8}$ Scale bar, 20 μm.

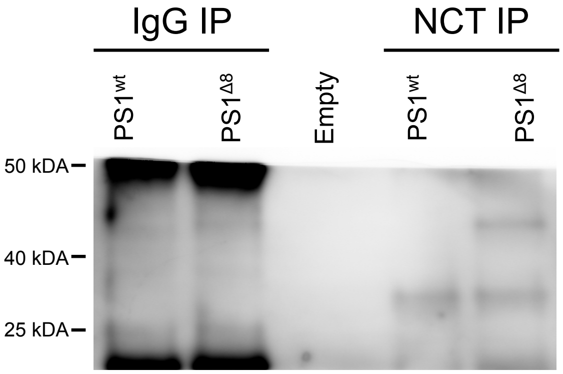

Supplemental Figure 2. Corroborative immunoblots from *in vivo* co- immunoprecipitation. Co-IP immunoblots of forebrain lysates of PS1$^{\Delta exon8}$-positive mice and controls. Blots made using a monoclonal anti- human PS1 specific antibody that detects full-length PS1 and PS1 NTF. Columns from left-to-right include immunoprecipitated lysates pulled using an Igg antibody IgG negative control (Rabbit IgG SC2027, Santa Cruz; Sheep anti-rabbit M-280 Dynabeads, Invitrogen), and NCT antibody (N1660, Sigma). Note that we only detect full-length PS1 (~45 kDA) in the mutant PS1$^{\Delta exon8}$ lysates (right lane). Also bands that likely represent PS1 NTF (~30kDA) are detected in the NCT immunoprecipitated lysates only, supporting that the IgG IP lysates do not interact with PS1 nonspecifically.
